# Growing old while staying young: The unique mechanisms that defy aging in plants

**DOI:** 10.1038/s44319-024-00062-4

**Published:** 2024-01-26

**Authors:** Darya Volkava, Karel Riha

**Affiliations:** Central European Institute of Technology (CEITEC), Masaryk University, Brno, Czech Republic

**Keywords:** Evolution & Ecology, Plant Biology

## Abstract

Although plants age like any other organisms, they have evolved to defy death for millennia and potentially forever.

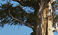

Aging is an inherent part of life that touches every living organism on our planet. It directly affects each individual as we witness how our bodies, health, and abilities change as we age. We experience a notable peak in physical performance at a certain age, followed by a gradual decline until death. Humans have long been captivated by the quest for the fountain of youth and unlocking the secrets of aging and longevity has fascinated scientists, philosophers, and many other individuals alike throughout the course of human history. It comes as no surprise that humans and animals have always been the primary focus of aging research and the theoretical framework of aging has been developed with these organisms in mind.

While there are very few animal species whose lifespan exceeds a century, some trees can live for millennia, making them the oldest living individuals on Earth.

Often overlooked in the field of aging research, however, are plants. This is unfortunate as plants can offer a unique and alternative perspective. While there are very few animal species whose lifespan exceeds a century, some trees can live for millennia, making them the oldest living individuals on Earth. In addition, and in striking contrast to animals where the risk of mortality typically increases and fertility decreases with age, perennial plants show only minimal signs of senescence, with some species even going in the opposite direction: decreased mortality and increased fecundity with age (Jones et al, 2014; Baudisch et al, 2013). This raises intriguing questions: Do plants age? How do they escape the typical trajectory of senescence, and what factors contribute to their remarkable longevity? What limits their lifespan? How are these unique characteristics intertwined with plant life strategies and evolutionary pathways?

## Senescence versus aging

There is no universal definition that encompasses all facets of aging across the tree of life. In comparative biology, aging can be defined as the time-dependent deterioration of an organism resulting in increased susceptibility to disease and environmental stress, age-related physiological changes, reduced fertility and increased mortality (Watson and Riha, 2011). When we work in the garden or walk through a meadow in the fall, we often observe plant decay, which is accompanied by general deterioration and eventual death. Intuitively, we associate these attributes with aging and are left with no other conclusion than that plants also experience aging. However, what we typically observe is senescence.

In plants, the term ‘senescence’ is used to describe a well-orchestrated developmental process designed to remobilize nutrients and minerals from vegetative tissues formed during the growing season into sink tissues, usually seeds (Thomas, 2013). This process supports energetically demanding seed maturation and reproductive success. In monocarpic plants, which flower only once during their lifetime, senescence is closely linked to reproduction and leads to the death of the organism. This is typical of annual plants that live only one season, but there are some long-lived monocarpic plants, such as bamboo, that can grow vegetatively for decades before flowering and eventually dying. Hence, senescence in plants should not be regarded as an aging process.

… senescence in plants should not be regarded as an aging process.

To begin with, senescence is not inherently linked to the death of the entire organism. For instance, autumn leaf senescence occurs reiteratively in many trees to recapture leaf nutrients and store them in the trunk and branches throughout the winter. These stored nutrients subsequently facilitate the regrowth of foliage and flowers in the following season. In many plants, senescence occurs concurrently with growth, with older leaves dying while new leaves are actively produced. Second, senescence is a highly regulated and orderly process governed by well-defined molecular pathways. Finally, leaf senescence is partially reversible. In a classical study, synthesis of the anti-aging phytohormone cytokinin in leaves from a senescence-induced promoter prevented leaf senescence in tobacco resulting in plants with evergreen foliage (Gan and Amasino, 1995). Thus, senescence in monocarpic plants can be viewed as the last step of a developmental program leading to the death of the entire body owing to massive mobilization of nutrients from vegetative parts to the seeds, rather than aging.

## Aging trees

If plant senescence is not considered as a typical form of aging, this brings us back to the question: Do plants age like animals? In a broad sense, aging results from the balance between two opposing processes: the inevitable accumulation of stochastic and environmentally induced damage to DNA, proteins, and other cellular and organismal components—which can be perceived as an increase in entropy or disorder—and the genetically encoded counteracting mechanisms such as repair, regeneration and renewal that mitigate and delay this damage at the cost of energy. In the context of antagonistic pleiotropy and disposable soma theories, aging is viewed as a trade-off between reproduction, self-maintenance, and longevity that is closely linked to an organism’s life strategy (Johnson et al, 2019).

Is there evidence to support the hypothesis that age-related physiological decline in plants limits their lifespan? Research has focused on this question primarily in relation to trees. This is not unexpected, as trees can be clearly defined in time and space and are able to survive long enough for changes in their physiology to be observed over time. Factors leading to tree mortality include both abiotic and biotic stresses, structural damage from natural disasters, competition for light, and human disturbance. Is tree aging a significant factor in increasing susceptibility to these stressors? This is a complex question, as there is little direct evidence to pinpoint the exact causes of tree mortality.

On the one hand, assessments of physiological, transcriptomic, and metabolomic parameters in old trees revealed an upregulation of stress response markers, suggesting that aging trees do experience physiological stress (Pasques and Munne-Bosch, 2023; Wang et al, 2020). As a tree increases in size, it must pump more water and nutrients from the soil to maintain its biomass, which can be limiting under conditions where adequate resources are not available (Mencuccini et al, 2005). The increasing distance between the roots and the canopy is a further constraint on water transport. These reasons may explain why tree growth slows with age. In addition, larger trees may be more susceptible to certain types of mechanical damage, such as lightning strikes or strong winds, especially if their structural integrity is compromised by pests or disease.

On the other hand, older and larger trees have more leaves, which can increase their photosynthetic activity and thus their production of energy and carbohydrates for growth and maintenance. Larger trees may also be more resilient due to their ability to store and access more resources and better withstand environmental stresses. Furthermore, old trees show no decline in meristem proliferation capacity and many other parameters of physiological performance, suggesting that wear and tear does not significantly affect their physiology (Pasques and Munne-Bosch, 2023; Wang et al, 2020). Compelling arguments for the view that trees and other perennial plants do not age come from demographic senescence studies that examine mortality and fertility patterns with age (Jones et al, 2014; Baudisch et al, 2013). Evolutionary theories of aging predict an increase in mortality risk and a decrease in fertility over the lifespan of multicellular iteroparous species, a pattern typically observed in most animals. However, an opposite pattern is seen in trees and other perennial plants. As plants grow larger, they produce more flowers and thus more seeds. Over time, they also develop a more robust structure that enhances their survival.

… old trees show no decline in meristem proliferation capacity and many other parameters of physiological performance, suggesting that wear and tear does not significantly affect their physiology.

## Methuselah

**Figure 1:**
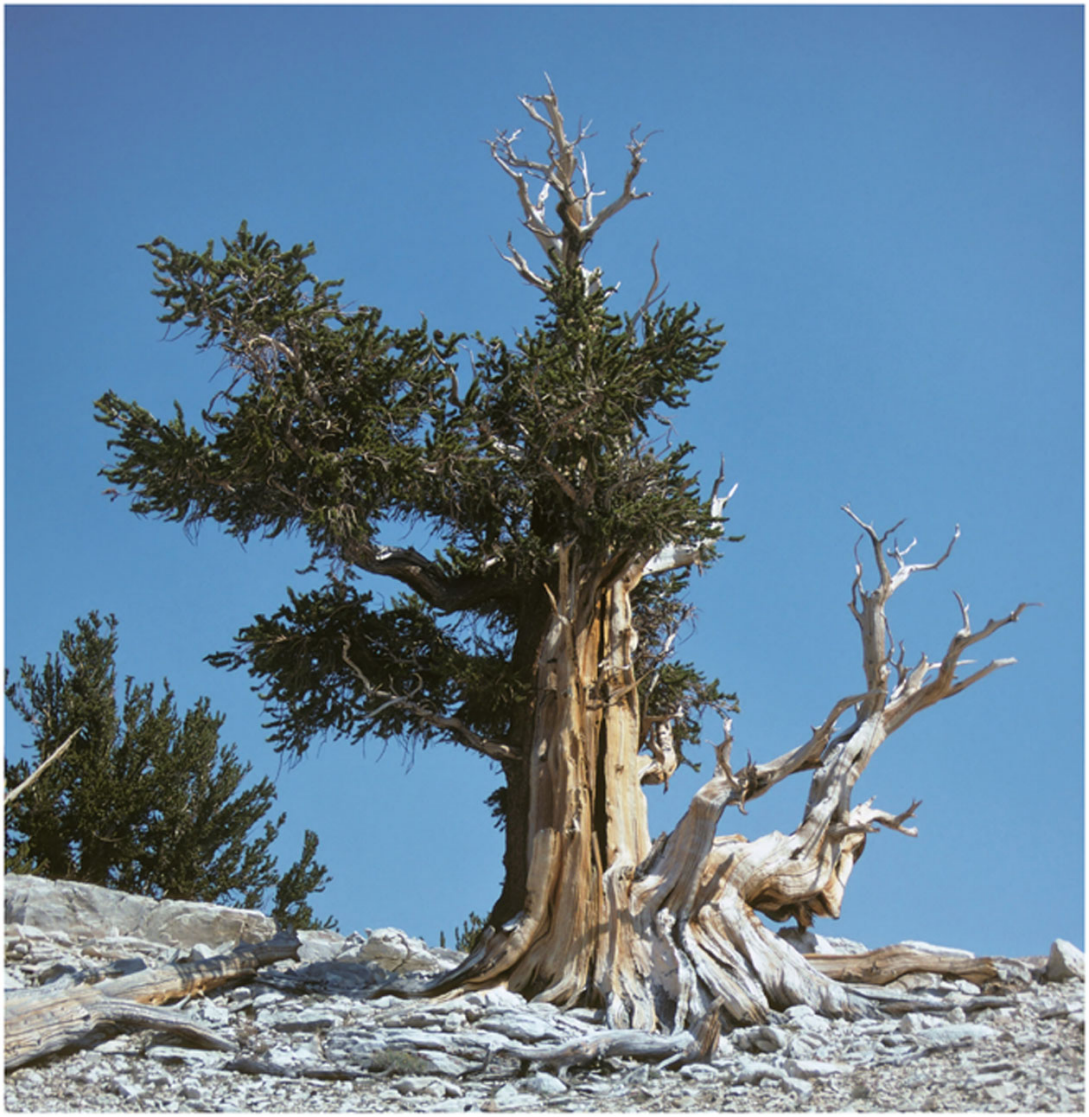
Ancient bristlecone pine *Pinus longaeva* in Patriarch Grove, White Mountains, California, USA. Wikimedia/Jim Morefield under a CC-SA 2.0 license.

Perhaps the best testament to a plant’s ability to defy aging is the age of some of the longest-lived plants themselves (Fig. 1). The oldest living tree, as well as the oldest living individual organism on Earth, is a bristlecone pine known as Methuselah, which is nearly 5000 years old. There are ~100 tree species the lifespans of which exceed 500 years, which is at the upper limit of the maximum lifespans of the quahog clam and the Greenland shark, the longest-living animals; about 30 tree species can grow for over a millennium (Liu et al, 2022). While woody plants typically outlive their herbaceous counterparts, there are also exceptions among herbs. *Borderea pyreneica*, an ephemeral herb in the Pyrenees Mountains, has a short growing season. It sprouts from a subterranean tuber in the spring, flowers and seeds rapidly, and dies above ground within a few weeks. Despite its ephemeral nature above ground, the tuber which produces new shoots each spring, survives for up to 300 years, earning the title of the oldest recorded herb (Munne-Bosch, 2007).

The most remarkable longevity records, however, belong to clonal plants, which form communities of genetically identical clones known as ramets. These communities, sometimes consisting of thousands of ramets linked through their root system to form a single super-organism, outlive individual senescence and death, and the entire clone can persist for many millennia. The Pando Forest in Utah, with 47,000 quaking aspen clones, is estimated to be between 14,000 and 40,000 years old. *Lomatia tasmanica*, an endangered Tasmanian shrub, originated from a single sterile triploid plant, and its colony has persisted through clonal propagation for at least 43,000 years (Thomas, 2013).

The Pando Forest in Utah, with 47,000 quaking aspen clones, is estimated to be between 14,000 and 40,000 years old.

## Plants’ lifehacks to defy aging

This extraordinary longevity leads to the question: what distinguishes plants from animals in their ability to persist over time? Features that contribute to the remarkable longevity of plants include indeterminate growth, a modular body plan, the seemingly limitless proliferative capacity of plant meristems, the pluripotency of plant somatic cells, and their ability to differentiate into germ cells. In addition, plants do not suffer from life-threatening malignancies and exhibit a disparity between cell death and the demise of the entire organism.

Animals have a predetermined body plan that is established during embryogenesis, and post-embryonic development is restricted to the enlargement and maintenance of pre-existing structures. This is achieved through the activity of adult stem cells, which are usually multipotent and can differentiate into a limited number of cell types. In contrast, plants are born as very rudimentary structures containing an embryonic root and an embryonic shoot, each of which harbors a cluster of pluripotent stem cells called the root apical meristem and the shoot apical meristem, respectively. All plant organs, including reproductive structures, are formed by indeterminate growth through continuous proliferation of meristematic cells throughout life. As long as the meristems remain active, the plant can continue to grow in size and complexity.

As long as the meristems remain active, the plant can continue to grow in size and complexity.

Multicellular organisms, such as plants and animals, are composed of multiple cells that work together in a coordinated manner. One of the challenges of multicellularity is maintaining proper control over cell division and growth. In animals, risk of malignant cancers that can spread throughout the body is one of the key limitations of longevity. However, plants do not suffer from malignant cancers. While deregulated cell proliferation can occur in plants, plant cells are surrounded and interconnected by rigid cell walls. Unlike in animals, where individual cells can migrate within tissues, plant cell walls constrain the movement and uncontrolled spreading of cells.

Another feature that distinguishes plants from animals is modular growth. The plant body is composed of repeating structural units, or modules, that can develop independently and often have the potential to function as separate entities (Munne-Bosch, 2007). Each module typically consists of stems, leaves, and reproductive structures. Modules are individually dispensable for plant survival and, if damaged or lost, can be replaced by differentiation of new units from intact meristems. In clonal plants, modules differentiated from horizontal branches or roots can give rise to new ramets and radially explore their environment for resources. Plants are sessile organisms, and the indeterminate and modular growth strategy allows them to adapt their physical structure to local environmental conditions. This developmental plasticity is a key element in their ability to survive and persist, as it allows them to renew or regenerate new structures in response to damage caused by disasters, pests, or disease.

Cell senescence and death is an inherent part of plant development. Living as a perennial plant is nothing more than a continuous cycle of cell death and regrowth. Tree biomass consists primarily of wood, the remains of cells that form a scaffold supporting a thin living layer of cells and organs produced by the activity of cambial and axillary meristems. As part of the constant renewal, most of the newly formed cells die and contribute to the formation of tree rings or are shed as senescent leaves or ripe fruits. This cyclic process ensures continuous regeneration of organs in subsequent seasons. Modular senescence is an important feature of the plant’s life strategy. It can be induced to remobilize and store resources to support regrowth or reproductive efforts, or triggered in response to pathogen attack to limit the spread of disease. Dead cells also play a central role as key structural elements that facilitate vertical plant growth and enhance access to sunlight. Thus, the orchestrated interplay between cell death and regrowth provides a strategy for responding to environmental challenges, ultimately enhancing the plant’s ability to thrive over extended periods of time.

## Meristems: the fountain of youth

Plant growth is driven by cell division in meristems. Meristems are relatively small clusters of cells, yet their proliferative activity is responsible for the formation of enormous amounts of cells. The entire Pando forest, consisting of 47,000 clonal trees with an estimated total weight of ~5.8 million kilograms, was generated by continuous cell divisions originating from two tiny clusters of meristematic cells in a single seed. This suggests that, unlike animals, plant stem cells do not undergo replicative senescence and have an unlimited capacity to proliferate. In animals, replicative senescence, the limited ability of somatic cells to proliferate, serves as an example of antagonistic pleiotropy. As cells divide and replicate their DNA, they increase the likelihood of mutations and cancer. Therefore, restricting cell proliferation through replicative senescence benefits an animal early in life by suppressing cancer, but impairs organ renewal later in life, contributing to aging. Plants, which are not susceptible to cancer, do not need to limit the proliferative capacity of their somatic cells. This characteristic greatly expands their ability to grow and clonally reproduce.

… unlike animals, plant stem cells do not undergo replicative senescence and have an unlimited capacity to proliferate.

However, the continuous proliferation of meristematic cells over centuries may lead to the accumulation of a significant number of mutations and, consequently, to a deterioration of cell physiology. Analysis of the reproductive performance of trembling aspen ramets derived from clonal colonies of different ages revealed a gradual decrease in pollen viability with increasing colony age (Ally et al, 2010). This suggests that extensive somatic growth may lead to mutations that are phenotypically manifested in haploid pollen. Nevertheless, there is no evidence to date to support the idea that somatic mutations play a significant role in physiological decline with age (Thomas, 2013).

**Figure 2:**
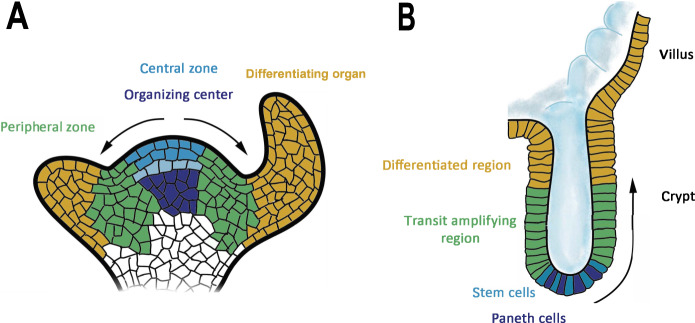
Comparison of plant and animal stem cell niches. (**A**) Shoot apical meristem with slowly proliferating cells in the central zone that are gradually displaced to the rapidly dividing peripheral zone. This gives rise to cells that will further differentiate into organs. (**B**) The crypt of the small intestine contains slowly proliferating stem cells at the base. Their division results in cell displacement into the transit amplifying region, where cells rapidly proliferate and give rise to cells that differentiate into intestinal epithelial cells. The direction of cell displacement is indicated by arrows.

Plants adopt several strategies to mitigate the risk of mutations. To begin with, plant stem cells appear to possess strict genome quality control mechanisms and robust DNA repair and genome maintenance (Watson and Riha, 2011). Another striking feature of plant stems cells is their infrequent division to minimize DNA replications, which is the major source of mutations (Watson et al, 2016). Shoot meristems have a stratified structure with a central zone containing quiescent or very slowly dividing stem cells that are surrounded by rapidly dividing cells in the peripheral zone (Fig. 2). Plant growth and tissue differentiation are driven by rapid divisions in the peripheral zone, whereas the central zone serves as a reservoir of cells with a low proliferation history that can repopulate the peripheral zone through occasional divisions (Watson et al, 2016). Such hierarchical organization of cell divisions in meristems can substantially reduce DNA replications and thus the risk of mutations in stem cells, while still supporting continuous growth. Interestingly, a similar organization of cell divisions occurs also in animal stem cell niches (Fig. 2) suggesting that plants and animals have independently invented the same solution to minimize mutations in stem cells. Finally, thanks to modular growth in plants, sectors carrying deleterious mutations (also called sports) can be effectively outcompeted and replaced by healthy modules.

## Can plants live forever?

Taken together, plants possess an impressive array of tools and mechanisms that seem to predispose them to immortality. Why, then, do ancient trees or immortal clones not dominate our world, since a longer lifespan should be an evolutionary advantage? On the contrary, truly ancient trees are extremely rare. They have been called “lottery winners” for their ability to live to an age 10–20 times longer than the average lifespan of their species, and they represent a tiny fraction of the population (Cannon et al, 2022). The majority of plants die much earlier due to extrinsic causes such as competition for light and resources, herbivory, disease, and other natural disturbances, including human intervention. The longer a plant lives, the greater the likelihood that it will eventually succumb to such events. Clonal propagation can provide a partial solution by spreading the risk over many clones and continuously generating new ones. However, this strategy also has limitations over long periods of time. Clonal plants form sessile, genetically homogeneous communities confined to specific areas, and changes in local environmental conditions that inevitably occur over millennia may exceed their adaptive potential (Ottaviani et al, 2022).

While there is ongoing debate about the maximum attainable human lifespan, it is obvious that our bodies are not designed to survive much beyond a century. Even with the best possible care and advances in biomedical technology, 150 years may be the upper limit. Plants, however, are built differently. Their life and developmental strategies seem to impose no limits on their theoretical lifespan. Plants can persist as long as they continually outgrow senescence and replace dying structures. To paraphrase Howard Thomas, a researcher devoted to plant senescence, “Aging is a fate that probably awaits all organisms; it’s just that plants are organized so that they’re not around when it happens” (Thomas, 2013). This leads to the question: can a plant live indefinitely under perfect conditions and with the greatest care? In fact, such experiments have already begun. Studies on trees have shown that extreme longevity correlates with slow growth, smaller stature, and nutrient-poor habitats (Liu et al, 2022; Piovesan and Biondi, 2021). Similar conditions are used in bonsai cultivation. With some of the oldest bonsai trees believed to be around 1000 years old, it is clear that this experiment has a long way to go.

Aging is a fate that probably awaits all organisms; it’s just that plants are organized so that they’re not around when it happens.

### Supplementary information

Peer Review File
