# Supplementary material for: Growing old while staying young: The unique mechanisms that defy aging in plants
Source: EMBO Rep. 2024 Jan 26;25(3):934–8. doi: 10.1038/s44319-024-00062-4 (PMC10933334; doi:10.1038/s44319-024-00062-4)
Supplement: Supplementary file 1 — Peer Review File [file 44319_2024_62_MOESM1_ESM.pdf]

EMBO reports

Growing old while staying young

Darya Volkava and Karel Riha
DOI: 10.15252/embr.202358581

Corresponding author(s): Karel Riha (Karel.Riha@ceitec.muni.cz)

**Review Timeline:**	Submission Date:	1st Dec 23
	Accepted:	10th Jan 24

Editor: Holger Breithaupt

Transaction Report: This article was editorially reviewed.
